# The efficacy and safety of minocycline as adjuvant therapy in refractory mycoplasma pneumonia in Chinese children: a meta-analysis

**DOI:** 10.1186/s13052-022-01362-y

**Published:** 2022-09-21

**Authors:** Hong-Xia Shen, Chang Liu, Hui-Jun Lin, Lu-Jie Xu, Guang-Yan Wang, Mei-Xing Yan

**Affiliations:** Department of Pharmacy, Women and Children’s Hospital, Qingdao University, No. 6 of Tongfu Street, Shibei District, Qingdao, 266034 China

**Keywords:** Minocycline, Mycoplasma pneumonia, Chinese children

## Abstract

**Background:**

To explore the efficacy and safety of minocycline as adjuvant therapy for refractory mycoplasma pneumonia in Chinese children.

**Methods:**

PubMed, EMBASE, Cochrane Library, CNKI, Wanfang database and VIP database were systematically searched. Studies where minocycline was used as adjuvant therapy for refractory mycoplasma pneumonia in Chinese children were included. The effect of numeration data and the measurement data were represented by odds ratios (OR) and weighted mean differences (MD), respectively. Review Manager version 5.3 was used to compare the treatment efficacy, time for the cough to subside, defervescence time, hospitalisation time, adverse events and other indicators.

**Results:**

Ten studies involving 857 patients were included in the final analysis. Compared with the conventional treatment of refractory mycoplasma pneumonia in children, the addition of minocycline as adjuvant therapy was found to improve the treatment efficacy (OR: 5.45; 95% CI: 3.46, 8.57, *p* < 0.001); shorten the duration of cough (MD: -3.61; 95%CI: -4.25, -2.97, *p* < 0.001), fever time (MD: -4.77; 95% CI: -6.30, -3.23, *p* < 0.001) and hospitalisation time (MD: -5.53 (95% CI: -7.19, -3.88, *p* < 0.001); and decrease the concentration of C-reactive protein (MD: -13.95; 95%CI: -18.61, -9.29; *p* < 0.001) and the erythrocyte sedimentation rate (MD: -10.88; 95% CI: -14.05, -7.72, *p* < 0.001). The use of minocycline did not lead to significant adverse events (OR = 0.63; 95% CI: 0.39, 1.01, *p* = 0.05).

**Conclusion:**

The use of minocycline as adjuvant treatment of refractory mycoplasma pneumonia in Chinese children has good efficacy and safety and may be promoted in clinical practice.

## Introduction

Mycoplasma pneumoniae is a common pathogen in respiratory tract infections and one of the most common causes of community-acquired pneumonia. Mycoplasma pneumonia is more prevalent in childhood and youth [1]. Previous epidemiological data show that its incidence is about 10–40% [2–4]. The incidence of mycoplasma pneumonia in children in China is 10–30% [5] and increases with age. In clinical practice, macrolide antibiotics such as erythromycin or azithromycin are often selected as the first-line treatment for mycoplasma pneumonia. However, due to the extensive use of macrolide antibiotics in recent years, the resistance of Mycoplasma pneumoniae to these drugs has gradually increased [3]. Mycoplasma pneumonia is difficult to alleviate in children in a short period of time. Pulmonary complications may arise, and refractory mycoplasma pneumonia may eventually develop. In vitro and in vivo studies have shown that tetracycline drugs still maintain strong antibacterial activity and clinical efficacy against Mycoplasma pneumoniae [6, 7], minocycline being one of them. In addition, previous studies have shown that the use of minocycline as adjuvant therapy in Mycoplasma pneumoniae infections can effectively improve the response to therapy and is safe. However, there is a lack of a systematic evaluation of the efficacy and safety of this drug, which makes its clinical promotion difficult due to insufficient evidence.

In China, resistant mycoplasma pneumonia is a serious public health problem that requires urgent attention. A previous study showed that an outbreak of macrolide-resistance mycoplasma pneumonia occurred in a primary school in Beijing, China [8]. The macrolide resistance rate was found to be 65.4% from 2014 to 2016, but the organism was susceptible to tetracycline and levofloxacin in vitro [9]. Although tetracyclines are contraindicated in children under eight years of age, it is now considered safe for short courses of treatment up to a certain dose. Therefore, the purpose of this study is to evaluate the efficacy and safety of minocycline as adjuvant treatment for refractory mycoplasma pneumonia in children in China, to provide a reasonable theoretical basis for the clinical use of minocycline.

## Materials and methods

### Search strategy

We followed the PRISMA guidelines for reporting systematic reviews and meta-analyses. A systematic literature search of PubMed, Embase, Cochrane Library, Web of Science, CINAHL, CNKI, the Wanfang database and the VIP database was performed from the date of inception of the databases to 30^th^ June 2022. A search strategy combining subject headings and free words was used. The keywords used in the English databases included ‘Minocycline’, ‘Mycoplasma pneumonia’, ‘China’, ‘Children’, ‘Safety’ and ‘Efficiency’. The search terms in the Chinese databases were the Chinese expressions for the same search keywords as in the English databases. Synonyms of each term were also used.

### Inclusion and exclusion criteria

The inclusion criteria were as follows: (1) a clinical diagnosis of mycoplasma pneumonia; (2) Chinese patients aged ≤ 16 years; (3) the use of minocycline adjuvant treatment for refractory mycoplasma pneumonia; (4) the control group received conventional treatment; and (5) the outcome indicators were treatment efficacy, treatment time and complications.

The exclusion criteria were as follows: (1) non-population studies; (2) conference articles, case reports and systematic reviews; (3) the outcome information was insufficient and could not be analysed; (4) duplicate reports of literature research.

### Study selection and data extraction

Two reviewers independently reviewed the abstracts and the full text of each article according to the inclusion and exclusion criteria. For disagreements between the two reviewers, a third reviewer was recruited for discussion until consensus was achieved. After literature screening, the two reviewers independently extracted the following information: demographic characteristics of the subjects, treatment methods for refractory mycoplasma pneumonia in children, treatment safety and effectiveness and outcomes.

### Assessments of methodological quality

Because this study only included RCTs, the Cochrane Collaboration tool for assessing the risk of bias was used to evaluate the quality of the literature. This tool is used to evaluate studies on various parameters, namely, the method of randomisation, allocation concealment, the method of blinding, the integrity of the data, whether there was selective reporting of results and other sources of bias.

### Statistical analysis

Review Manager version 5.3 (Revman 5.3) software was used for statistical analysis. The effect of the numerical data and the measurement data was expressed by the odds ratio (OR) and the mean difference (MD). The 95% confidence interval (CI) was used to estimate the interval range of the effect. The I^2^ statistic was used to determine the degree of heterogeneity. If $\mathrm I^{2\;}\;\mathrm{was}\;\mathrm{less}\;\mathrm{than}$ 50% or *p* > 0.1, the included literature was considered homogeneous, and the fixed effect model (Mantel–Haenszel) was used for analysis. If I^2^ was more than 50% or *p* ≤ 0.1, the included studies were considered heterogeneous, and the random effect model (DerSimonian–Laird) was used for analysis. If heterogeneity was high, sensitivity analysis was used to explore the source of heterogeneity. A *p* < 0.05 indicated that the difference was statistically significant.

## Results

### Study characteristics

**Fig. 1:**
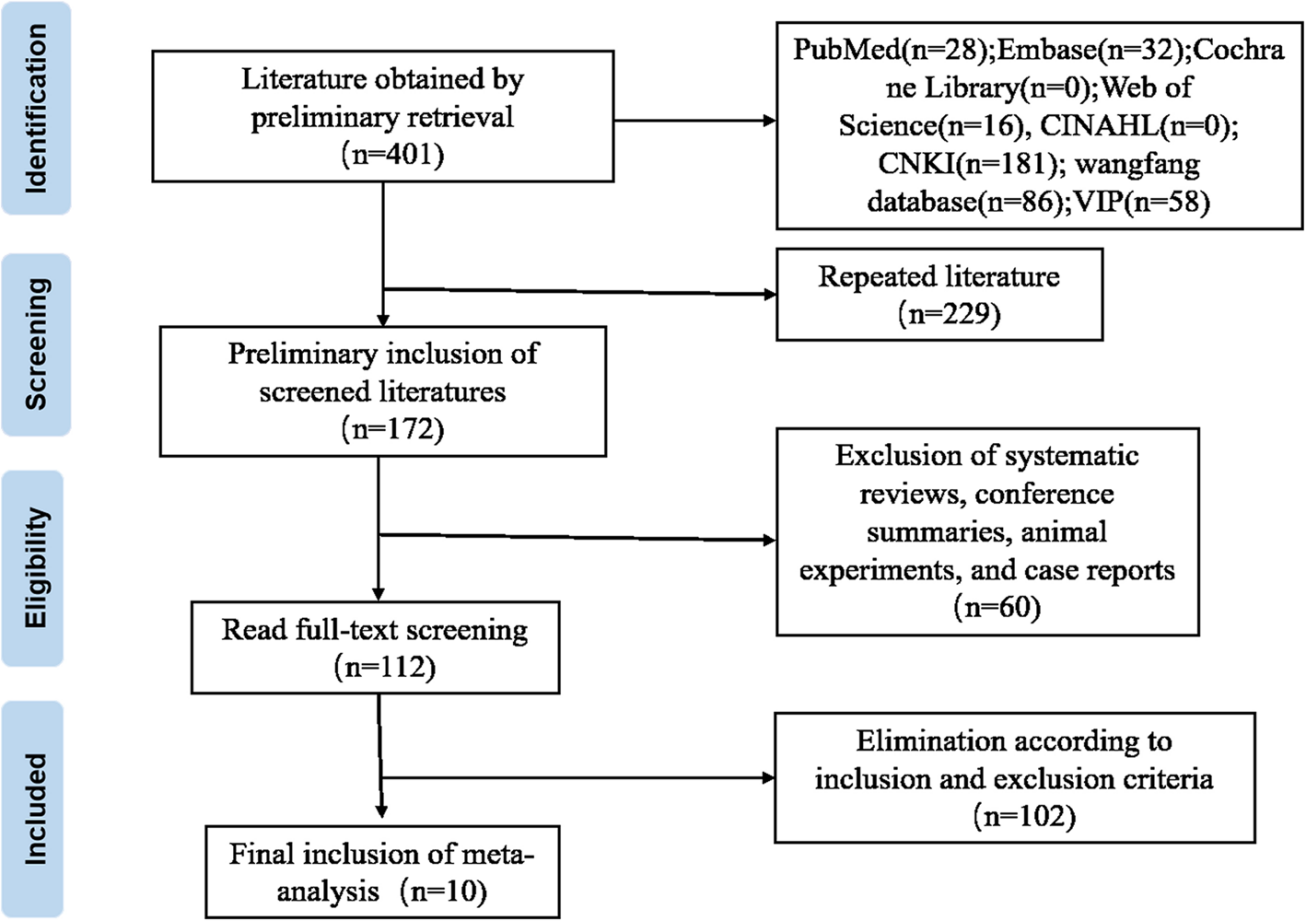
Flow chart of literature selection

A total of 10 articles were included in this study after systematic retrieval and screening of Chinese and English databases [10–19]. The literature screening process is shown in Fig. 1. The 10 studies involved 857 Chinese children with refractory mycoplasma pneumonia. Among them, three studies reported the course of the disease, two studies used the conventional treatment of erythromycin and azithromycin alternately and eight studies only used the conventional treatment of azithromycin. The duration of treatment for five studies was one week and four weeks for three studies. Another two studies ended the dosing two days after the fever subsided. See Table 1.

**Table 1:** Characteristics of the included studies

Study	Sample size (C/E)	Age (C/E; y)	Man (%)	Course of disease (C/E; d)	Treatment (C/E)	Therapeutic time(week)
Wang Bo,2013	35	10.3 ± 3.9	36(51.4)	NR	azithromycin10mg/Kg·d; minocycline4mg/Kg·d	1
	35	9.4 ± 4.5			azithromycin10mg/Kg/d	
Yi Qiaoling,2013	28	12.3 ± 3.8	29(51.8)	NR	erythromycin、azithromycin30-50 mg/Kg·d; minocycline4mg/Kg·d	1
	28	12.6 ± 3.9			erythromycin、azithromycin30-50 mg/Kg·d	
Zheng Qingkai,2018	40	11.1(8–14)	48(60.0)	NR	azithromycin10mg/Kg·d; minocycline4mg/Kg·d	1
	40	10.7(7–13)			azithromycin10mg/Kg·d	
Liang Zou,2016	23	11.4 ± 2.7	25(54.3)	NR	azithromycin2mg/mL·d; minocycline4mg/Kg·d	1
	23	12.5 ± 2.6			azithromycin2mg/mL·d	
Chen Ying,2012	20	11.6 ± 2.1	19(47.5)	NR	erythromycin、azithromycin; minocycline4mg/Kg·d	1
	20				erythromycin、azithromycin	
Wang Yongxia,2013	19	10.47 ± 1.98	20(54.1)	NR	azithromycin10mg/Kg·d; minocycline4mg/Kg·d	4
	18	10.41 ± 1.98			azithromycin10mg/Kg/d	
Rao Fuguang,2016	44	8.9 ± 1.7	51(58.0)	2.4 ± 0.9	azithromycin10mg/Kg·d; minocycline4mg/Kg·d	4
	44	8.6 ± 2.2		2.2 ± 0.7	azithromycin10mg/Kg/d	
Huang Wei,2018	40	6.51 ± 1.50	49(61.3)	12.30 ± 1.25	azithromycin10mg/Kg·d; minocycline4mg/Kg·d	4
	40	7.62 ± 1.41		13.12 ± 1.06	azithromycin10mg/Kg/d	
Sun Xiangyang,2016	120	9.6 ± 1.9	58(48.3)	5.8 ± 1.2	azithromycin10mg/Kg/d, minocycline 50 mg, twice a day	Discontinue 2 days after fever subsides
	120	9.8 ± 2.0	56(46.7)	6.0 ± 1.3	azithromycin10mg/Kg/d	
Xu Xiaohong,2017	60	9.2 ± 2.1	32(50.3)	NR	azithromycin10mg/Kg/d, minocycline 50 mg, twice a day	Discontinue 2 days after fever subsides
	60	8.9 ± 2.4	33(55.0)		azithromycin10mg/Kg/d	

*C* Control group, *E* Experimental group, *NR* Not reported

### Literature quality evaluation

**Fig. 2:**
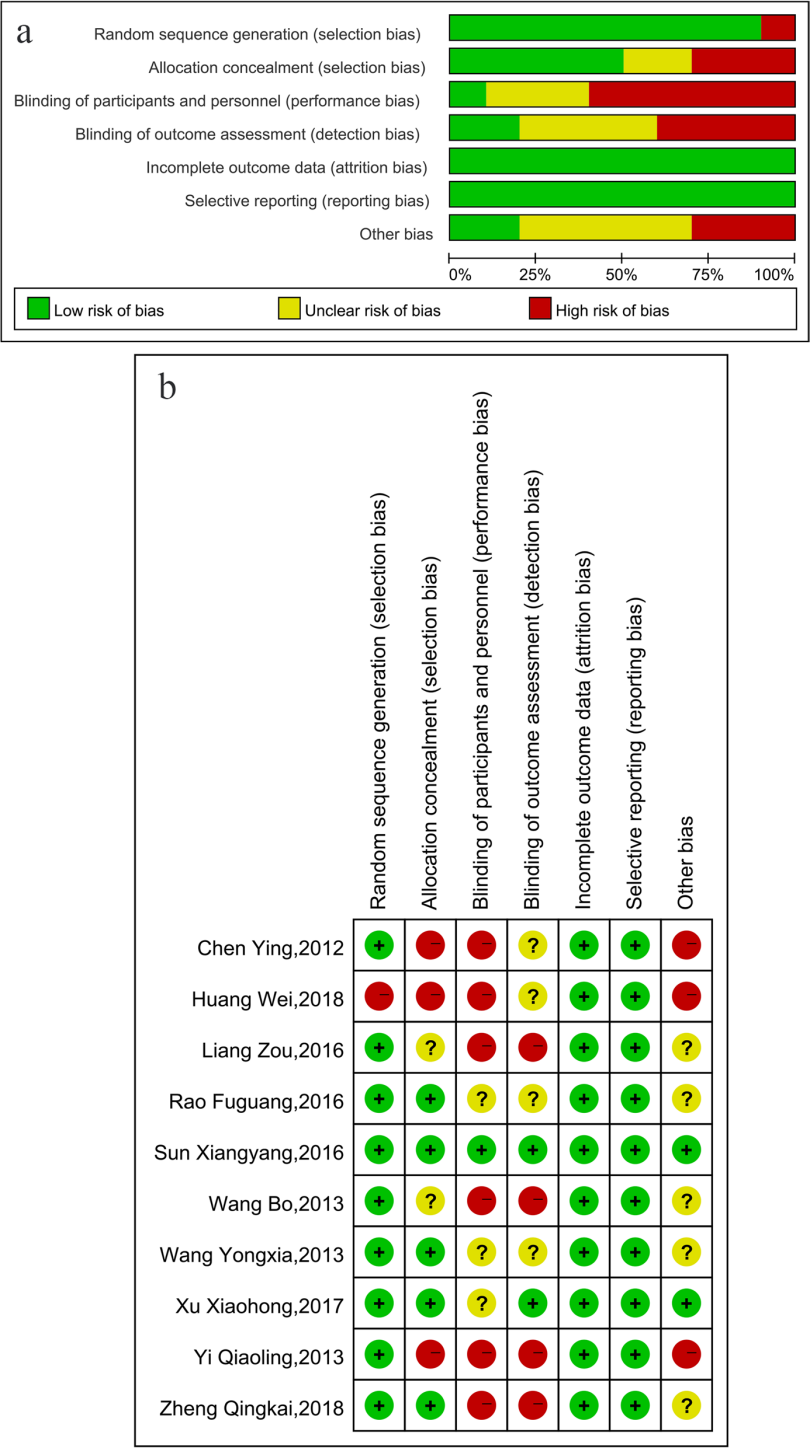
a. Risk of bias graph included in 10 studies; b. Risk of bias summary of included in 10 studies

Quality assessment using the Cochrane Collaboration risk of bias assessment tool found that the risk of poor data integrity and selective reporting in the included studies was low, but the risk of bias in the implementation of blinding methods was high. Figure 2 shows the results of the detailed quality evaluation.

### Treatment efficacy

**Fig. 3:**
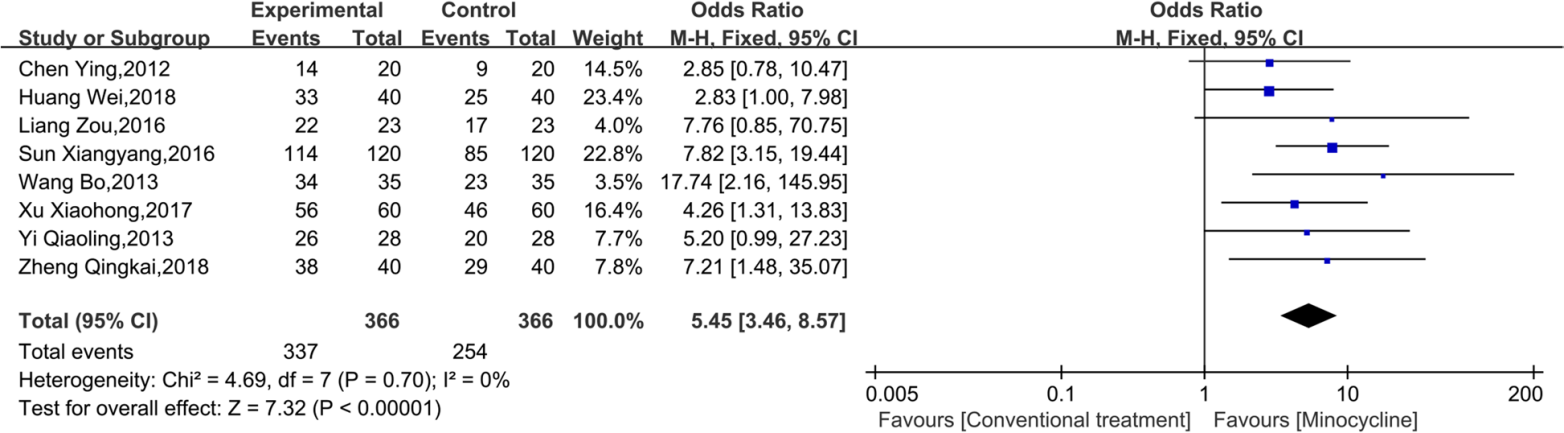
Meta-analysis of the efficacy of minocycline in adjuvant treatment of refractory mycoplasma pneumonia in Chinese children

Eight studies reported the efficacy of minocycline as adjuvant treatment in refractory mycoplasma pneumonia in Chinese children. The heterogeneity test showed an I^2^ = 0% and *p* = 0.70, suggesting good homogeneity among the included studies. The fixed effect model was hence used for analysis. The results of the meta-analysis showed that 366 people were in the observation group and the control group, respectively. In addition, minocycline improved the response to treatment to a certain extent. The combined effect OR was 5.45 (95% CI: 3.46, 8.57, *p* < 0.001), as shown in Fig. 3.

### Time for the cough to subside

**Fig. 4:**
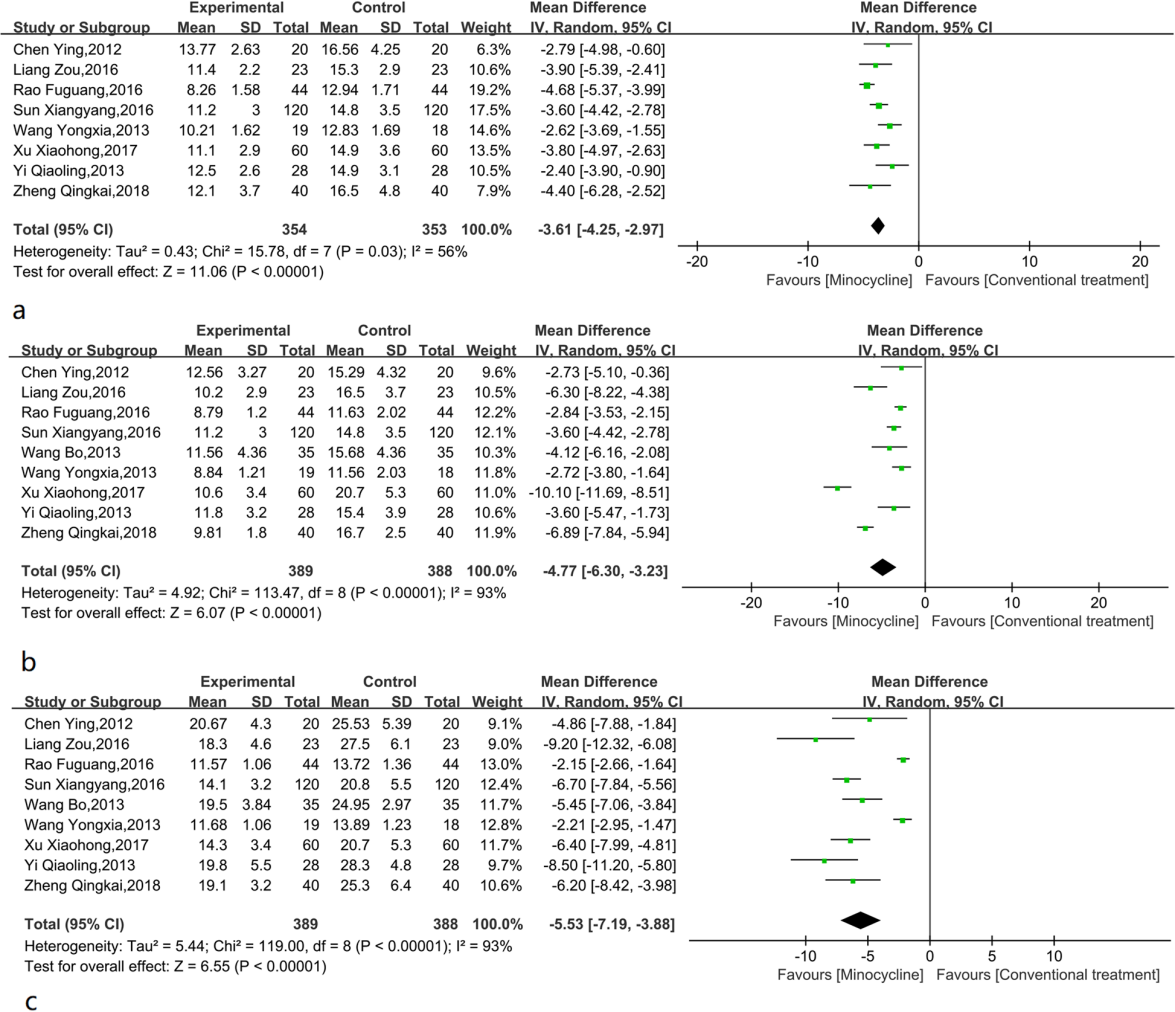
**a**. Meta-analysis of cough subsided time (**d**) of minocycline-assisted treatment for refractory mycoplasma pneumonia in Chinese children; **b**. Meta-analysis of antipyretic time (**d**) of minocycline adjuvant therapy for refractory mycoplasma pneumonia in Chinese children; **c**. Meta-analysis of hospitalization time (**d**) of minocycline-assisted treatment for refractory mycoplasma pneumonia in Chinese children

Eight studies reported the time for the cough to subside. Analysis showed that the heterogeneity of studies included in the analysis was large (I^2^ = 56%, *p* = 0.03). According to the meta-analysis results, minocycline could effectively shorten the time for the cough to subside compared with the control group (Fig. 4a). Compared with the control group, the time for the cough to subside was shortened by -3.61 (95% CI: -4.25, -2.97, *p* < 0.001) days by using minocycline. Through sensitivity analysis, heterogeneity was significantly reduced after excluding one study [16], (I^2^ = 4%, *p* = 0.40). After using the fixed effect model, the combined effect was -3.36 (95% CI: -3.84, -2.89, *p* < 0.001) days.

### Defervescence time

Nine studies reported the results of time for defervescence. Analysis showed that the heterogeneity between studies was large (I^2^ = 93%, *p* < 0.001). The random effect model was used for meta-analysis. Meta-analysis of the defervescence time (Fig. 4b) showed that minocycline reduced defervescence time by about -4.77 (95% CI: -6.30, -3.23, *P* < 0.001) days compared with the control group. There was no clear source of heterogeneity identified in the sensitivity analysis, suggesting a relatively stable heterogeneity between included studies.

### Hospitalisation time

Nine studies reported the results of hospitalisation time. The heterogeneity test results were I^2^ = 93% and *p* < 0.001, suggesting that the heterogeneity among the included studies was high, and the random effect model was hence selected for analysis. Figure 4c shows the effect of minocycline adjuvant therapy on hospitalisation time in children with refractory pneumonia. The use of minocycline adjuvant therapy shortened the hospitalisation time by -5.53 (95% CI: -7.19, -3.88, *p* < 0.001) days. After the sensitivity analysis, two studies [15, 16] were excluded. The heterogeneity test now showed an I^2^ = 24%, and *p* = 0.24, and a combined effect of -6.51 (95% CI: -7.20, -5.82, *p* < 0.001) days.

### Inflammatory factors

**Fig. 5:**
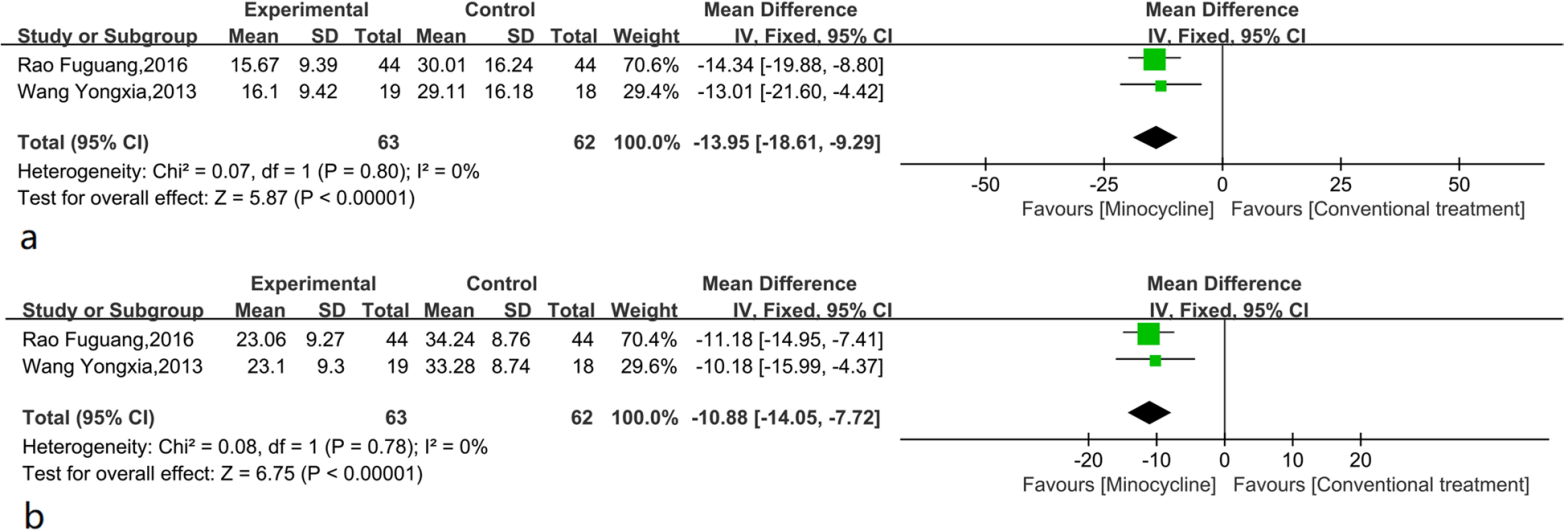
**a**. Meta-analysis of CRP (mg/L) in minocycline adjuvant therapy for refractory mycoplasma pneumonia in Chinese children; **b**. Meta-analysis of ESR (mm/L) in minocycline adjuvant therapy for refractory mycoplasma pneumonia in Chinese children

Two studies reported the results of the inflammatory factor C-reactive protein (CRP) and erythrocyte sedimentation rate (ESR) after minocycline treatment. The included studies had good homogeneity (I^2^ = 0%), and the fixed effect model was selected for analysis. Compared with the control group, after minocycline treatment, CRP significantly decreased by -13.95 (95% CI: -18.61, -9.29, *p* < 0.001) mg/L, and the difference was statistically significant (Fig. 5a). In addition, minocycline could effectively reduce ESR by -10.88 (95% CI: -14.05, -7.72, *p* < 0.001) mm/hr (Fig. 5b).

### Adverse events

**Fig. 6:**
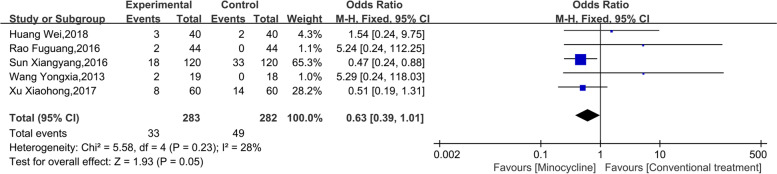
Meta-analysis of adverse events in minocycline adjuvant therapy for refractory mycoplasma pneumonia in Chinese children

Five studies reported adverse events caused by minocycline treatment. The heterogeneity test suggested that heterogeneity between the studies was low (I^2^ = 28%, *p* = 0.18), and the fixed effect model was used for analysis. The results of the meta-analysis (Fig. 6) showed that compared with the conventional treatment, the difference in adverse events caused by minocycline-assisted treatment in children with refractory mycoplasma pneumonia was not statistically significant, and the combined effect OR was 0.63 (95% CI: 0.39, 1.01, *p* = 0.05).

### Sensitivity analysis

Minocycline is not usually used in children under eight years of age. The mean age of the subjects in one study was less than eight years, so we further conducted a sensitivity analysis. The results showed that minocycline still had a positive effect on improving the treatment efficacy (OR = 6.25, 95% CI:3.75, 10.40; *p* < 0.001). The results of the analysis of adverse events showed that the use of minocycline had no significant effect. The combined effect was 0.58 (95% CI:0.36, 0.96; *p* = 0.03).

To exclude the influence of methylprednisolone on the results, further sensitivity analyses were carried out. After excluding three studies, the sensitivity analyses results showed that the use of minocycline as adjuvant treatment of refractory mycoplasma pneumonia could still effectively improve the treatment efficacy (OR = 6.25; 95% CI: 3.75, 10.40), shorten the time of fever (MD = -5.39; 95% CI: -7.28, -3.50), shorten the time for the cough to subside (MD = -3.55; 95% CI: -4.07, -3.02) and hospitalisation time (MD = -6.51; 95% CI: -7.20, -5.82). Besides, the difference between the two groups was not statistically significant (OR = 0.68; 95%CI:0.29, 1.61; *P* = 0.38).

#### Publication bias

**Fig. 7:**
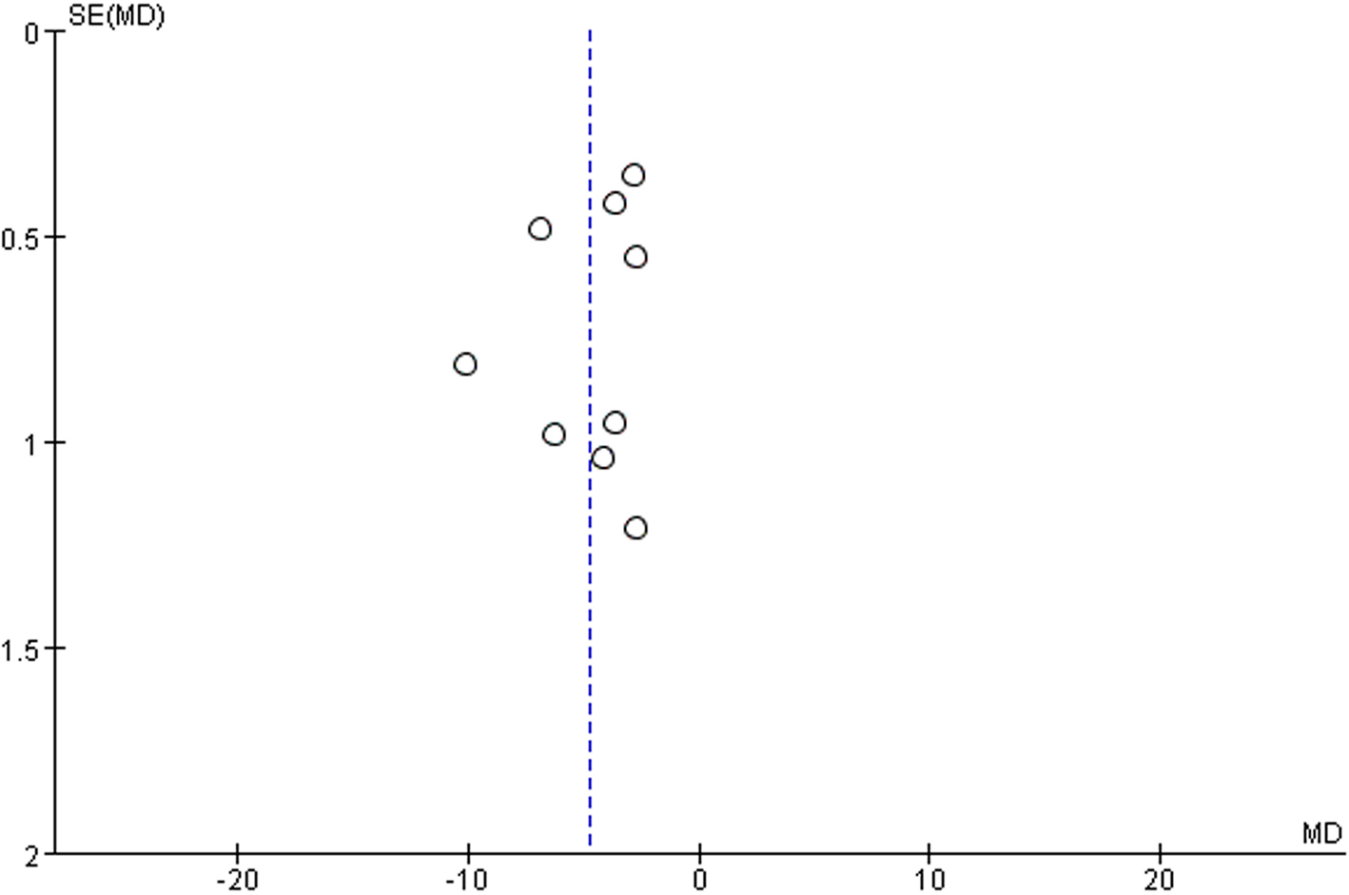
Funnel plot of publication bias

The results of the analysis for publication bias showed that the scattered points in the funnel plot were evenly distributed on the left and right sides, suggesting that the publication bias between the included studies was small and acceptable, as shown in Fig. 7.

## Discussion

The results of this study show that the use of minocycline as adjuvant therapy with routine treatment of mycoplasma pneumonia in children can effectively improve the treatment efficacy, suggesting that the use of minocycline is protective and results in benign treatment outcomes. In addition, it has a significant positive effect on the improvement of clinical symptoms. The results show that after minocycline treatment, the duration of cough and fever is shortened by about 3.5 days. Moreover, inflammatory factors such as CRP and ESR are significantly reduced, which effectively shortens the hospitalisation time.

Although macrolides are the first-line drugs for mycoplasma pneumonia and corticosteroids have a beneficial effect on treating refractory mycoplasma pneumonia [20], there are reports of drug resistance of Mycoplasma pneumoniae to macrolides all over the world [21]. In China, the rate of macrolide-resistant Mycoplasma pneumoniae is very high, reaching 54.5–100% [22], which makes it difficult to control the disease in a short time. In response to this situation, tetracycline drugs represented by minocycline are particularly important. A single-centre retrospective study [23] showed that the early use of minocycline could safely prevent and treat refractory mycoplasma pneumonia not responsive to macrolides. A systematic review showed that minocycline was a reasonable substitute for doxycycline in the following scenarios: skin and soft-tissue infections and outpatient treatment of community-acquired pneumonia in young [24]. In addition, the Japanese Guidelines for the Management of Respiratory Infectious Diseases in Children recommend the use of tetracycline antibiotics if clinical symptoms do not subside after 48 h of macrolide treatment [25]. Studies have shown that minocycline has a positive antibacterial activity against Mycoplasma pneumoniae [22, 26]. Further, clinical results confirm that minocycline has a positive effect on the treatment of refractory mycoplasma pneumonia in children [27–30]. However, although minocycline has a positive therapeutic effect on mycoplasma pneumonia, it is still necessary to be alert to the safety of tetracyclines and strictly abide by the application principles of antibiotics.

This study has some limitations. First, after a systematic search and screening, only 10 studies were included in the final meta-analysis, suggesting a lack of evidence in this field in China. Second, the sample size of the included literature is small, and the sample size exceeded 50 in only one study, which makes the sample data less representative. Third, the original research is based on the hospitals where the researchers work. A lack of multi-centre research in the choice of study subjects may have a potential selection bias. Finally, the differences in age, course of disease and treatment time of the subjects resulted in high heterogeneity in some of the analyses. Although heterogeneity is effectively reduced after sensitivity analysis, we still need to be vigilant about the impact of heterogeneity.

## Conclusions

In summary, compared with the conventional treatment of refractory mycoplasma pneumonia in children, the addition of minocycline as adjuvant therapy can effectively improve the treatment efficacy, shorten the duration of cough, fever, and hospitalization, reduce the concentration of inflammatory factors, and does not lead to obvious adverse events. The results of this study suggest that the use of minocycline can be promoted in clinical practice. However, considering the limitations of this study, more large-scale multicenter studies involving multiple races are needed to confirm the conclusions of this study.

## Data Availability

All data generated or analyzed during this study are included in this published article.

## Abbreviations

OR: Odds ratios
MD: Mean differences
CRP: C-reactive protein
ESR: Erythrocyte sedimentation rate
